# Development and initial validation of the Partnership Scale-DanceSport Couples

**DOI:** 10.3389/fpsyg.2023.1032767

**Published:** 2023-02-23

**Authors:** Xiuxia Liu, Guan Yang, Shen Wang, Xiangfei Wang, Xuelian Wang

**Affiliations:** ^1^Post-Doctoral Research Station, Wuhan Sports University, Wuhan, China; ^2^School of Physical Education, South China University of Technology, Guangzhou, China; ^3^School of Physical Education and Sport Science, Fujian Normal University, Fuzhou, China; ^4^School of Journalism and Communication, Wuhan Sports University, Wuhan, China

**Keywords:** partnership, DanceSport, scale development, athlete, obligatory instrumental ties, expressive ties, interpersonal perception

## Abstract

**Introduction:**

DanceSport is described as a dance involving a male–female partner. It is important to comprehend the partnership between dance couples so that their competitive performance can be effectively supported. However, only a few studies have verified the influence of partnership between DanceSport couples on competitive performance and explored its psychological mechanism to provide means to deal with the partnership. The reason was that there was a lack of appropriate assessment tools.

**Aims:**

This multi-study outlines the development, content, and construct validity of a novel, mixed-method tool to assess DanceSport partnership.

**Methods:**

The development of the Partnership Scale-DanceSport Couples (PS-DSC) included four studies and data from four samples of Chinese elite dancers (*N* = 914 total). In stage 1, outlined in study 1, PS-DSC items were generated and then refined using the feedback provided by academics, sports coaches, and elite dancers. In stage 2, outlined in studies 2 and 3, exploratory factor analysis and confirmatory factor analysis were used to examine the structure of the PS-DSC items. In stage 3, outlined in study 4, composite reliability, discriminant validity, and convergent validity were assessed. The result of this process was a 13-item three-factor instrument. Based on these initial findings, the PS-DSC provided the first valid and reliable way of measuring partnerships between DanceSport couples.

**Conclusion:**

This study has taken the promising first step in developing a tool to comprehensively measure partnerships between DanceSport couples.

## 1. Introduction

DanceSport (also called ballroom dance/competitive dance) is an extremely complex sports discipline (Vermey, 1994) that contains ten kinds of dances, namely, Cha, Samba, Rumba, Paso Doble, Jive, Waltz, Tango, Viennese Waltz, Slow Foxtrot, and Quickstep. Starting as one of the social graces expected of the European upper classes, DanceSport derives from the Latin word “balare” (to dance) and is now an umbrella term for a style of elite partner dancing (Ogilvie, 2017). This kind of partner dancing is danced both competitively (Bulman, 2006; Bria et al., 2011) and socially (Marion, 2006), in which there is a phenomenon of “feeling your partner” (Fostiak, 1996).

DanceSport requires partners to follow the rhythm of the music and compete against other dance couples to display the beauty of the sport (Jae Sung and Yoo Bin, 2012). Therefore, partnering skills are one of the important judging components in World DanceSport Federation competitions (Remelč et al., 2019; Yoshida et al., 2020). The overwhelmingly dominant aesthetic of DanceSport is the pairing cooperation of the male and female (Marion, 2006; Budnik-Przybylska et al., 2015). The prerequisite for demonstrating the beauty of this duet is having a partner; however, it is not easy to establish, maintain, and develop a partnership as different dancers have various life backgrounds that shape diverse personalities and habits and hence the conflicts in the process of interaction are unavoidable. In addition, once a partnership is established, due to the need for competition and training, the dancers face a longer period of interaction with their partners and their lives. Training and competition are highly intertwined (Reynolds, 1998) that a relationship of overlapping instrumental ties and expressive ties will be formed between partners (Wang, 2018), the boundary between the instrumental ties (business-like partnerships) and expressive ties becomes more blurred, and it is easy to break the partnership because of love issues (Liu et al., 2022). Therefore, for the dancers, besides the great amount of practice (diligence and endurance) and a high level of technical expertise (for which a good trainer is important), their partners are vital, and good cooperation between partners is of decisive importance for effective competition (Peters, 1992; Majoross et al., 2008; Budnik-Przybylska et al., 2015). Some scholars even argued that partnership was the essential characteristic of DanceSport that distinguishes it from other sports (Marion, 2006; Ogilvie, 2017). Therefore, a number of coaching hours of DanceSport go into attempting to depict the performing “male/female relationship” between partners (Marion, 2006). However, what is the nature of the DanceSport partnership? How to examine the partnership between DanceSport couples? Few studies have been conducted to address these issues, which would have the consequence of failing to advance the practice and theory of DanceSport at the interpersonal level. In view of this, Majoross et al. (2008) suggested that a new method should be developed to obtain a more elaborate and precise picture of this special connection. Based on this, we hoped to address this issue with the Partnership Scale-DanceSport Couples (PS-DSC) developed in this study. In practice, coaches and elite dancers can use the scale to assess the quality of a partnership. The higher the scores on each dimension, the stronger the motivation to excel in competition and the stronger the partnership. In terms of theoretical research, researchers can use the scale to measure partnerships to advance research on the relationship between partnerships and other variables, which will help guide related practice.

### 1.1. The concept and framework of DanceSport partnership

The development of a DanceSport partnership scale is an effective way to obtain information about personal relationships between elite dancer couples. Through a literature search, it was found that there were a few articles by researchers from 16 countries (Spain, India, South Korea, Hungary, USA, Canada, UK, Romania, France, Germany, Slovenia, Switzerland, Lithuania, Poland, Israel, and China) focusing on DanceSport partnerships. Among them, only Korean scholars have extracted the partnership dimension and developed assessment tools (Myung et al., 2010; Kim et al., 2011). Myung et al. (2010) studied the DanceSport partnership on grounded theory, conceptualized the DanceSport partnership as “a bilateral relationship based on mutual trust, sharing interests and risks among dance couples and assuming equal responsibility for achievements” based on the survey of 389 Korean elite dancers, and determined the 5 dimensions of partnership, namely, *partner care, partner harmony, reciprocal endeavor, perfect rhythm, and economical environment*. *Partner care* refers to being patient and considerate of dance partners and trying not to feel uncomfortable when getting along. *Partner harmony* refers to understanding the personality of the partners, being good at communication, and connecting with their hearts. *reciprocal endeavor* requires a cooperative attitude to pay earnest efforts to train frequently. *Perfect rhythm* refers to an intense sense of music and technical tension. *Economical environment* refers to having certain economic conditions and affording training and competition because the achievements of dancers are based on inviting teachers to attend classes for a long time and expensive clothing, among others. Based on Myung et al. (2010), Kim et al. (2011) found that the DanceSport partnership was a cooperative relationship that has the characteristics of mutual understanding and cooperative spirit in four dimensions, namely, *trust, referee-audience feedback, physical harmony, and economic conditions.* Among them, *trust* is the basic requirement for a partnership; *referee-audience feedback* is the demand of partners’ performance; *physical harmony* indicates that the dancers need to be harmonious in appearance, physique, body proportion, and body shape with the partners; and *economical environment* is considered the same as that mentioned in the study of Myung et al. (2010). In addition, Kim et al. (2011) also took 323 SportDancers registered with the Korean DanceSport Association for the survey, further verified the concepts and dimensions of the partnership, and developed the Korean DanceSport partnership scale, which contained 18 items utilized by other studies (Jae Sung and Yoo Bin, 2012; Yang, 2016).

Overall, the above four-dimensional theory and five-dimensional theory still have the following 3 limitations. (1) As a kind of interpersonal relationship, the psychological connection between DanceSport partners will be an important object of attention. However, *economical environment* and *physical harmony* are the influencing factors of the formation and maintenance of the partnership; *perfect rhythm* is the influencing factor of partner cooperation ability at the individual level, and *referee-audience feedback* is the influencing factor affected by the competitive performance of the partners. These are difficult to reflect the psychological connection between the partners and are difficult to regard as the dimensions of the partnership. (2) There are a few dimensions of partnership belonging to different disciplines or under different theoretical perspectives, such as sociology and musicology, which are not conducive to dialogue with relevant theories. As a special interpersonal relationship, it is the research concept of sports psychology. Although two representative studies have also found trust, partner care, partner harmony, and reciprocal endeavor from the perspective of sports psychology, partner care, partner harmony, and mutual assistance overlap to a certain extent, and hence, independence between the dimensions is weak. (3) DanceSport is a discipline based on romantic fantasy, which openly expresses emotions (Harman, 2019), shows the body between men and women, and highlights the intimate relationship between them (Ericksen, 2011). Therefore, compared with other dyadic sport events, such as ping-pong mixed doubles, tennis mixed doubles, and synchronized diving, DanceSport partnership is significantly different (Myung et al., 2010; Jae Sung and Yoo Bin, 2012). However, in the existing research on the partnership between DanceSport couples, due attention has not been paid to its characteristics, such as short-term passion. (4) Interpersonal relationship has cultural characteristics (Hsu, 1953) and so does DanceSport partnership. For example, based on the Rasch model in item response theory (IRT), Yang (2015) found that “my partner and I will be full of vitality when dancing in front of the crowd” and “my partner and I have left a deep impression on the audience” (Kim et al., 2011) were considered to be prejudiced against the male group because South Korean men cannot be regarded as the object of appreciation. However, the scale developed under specific cultural backgrounds should not be used uncritically in different cultures (Duda and Hayashi, 1998). However, few studies have paid attention to the cultural characteristics of DanceSport partnership from the perspective of cultural psychology. For countries including China based on Confucius culture, the obligations between DanceSport couples are ruled by “*renqing*” which focuses on the partnership itself without always considering that the instrumental intentions have not been recognized.

### 1.2. DanceSport partnership in our study

DanceSport partnership encompasses behaviors, emotions, and thoughts between two people (Kelley et al., 1983). In addition, according to the epistemological strategy of cultural psychology, that is, “one mind, many meanings; disunity universalism” (Shweder et al., 1998: 871; Cheung et al., 2011; Hwang, 2018), as well as the existing research results of DanceSport partnership and dance partner interaction practice, DanceSport partnership has a cultural identity. Based on these perspectives, we propose that the partnership between Chinese DanceSport partners includes obligatory instrumental ties (behaviors), expressive ties (emotions), and interpersonal perception (thoughts).

**Obligatory instrumental ties** (OIT) refer to the reciprocal behavior tendency of elite dancer couples based on the principle of obligation ruled by “*renqing*” to maintain their partnership during the whole process of taking DanceSport competition as the goal. It includes the obligation and instrument factors (Liu et al., 2022). First, because the importance of effective talent development in sports is well established as a key aspect of achieving high-level performance (Taylor et al., 2022), the competitive victory in DanceSport is taken as the starting point (Bednarzowa and Młodzikowska, 1983; Budnik-Przybylska et al., 2015). In addition, according to Chen et al. (2013), the analysis of the characteristics of Chinese “guanxi (interpersonal relationship) will always be considered as a capital,” as it can be found that the DanceSport partnership is a tool to achieve goals. Therefore, partners often exhibit a high degree of reciprocity and interaction (Reisman, 1981; Marion, 2006), and partnership is always taken as an instrument to obtain benefits (Lai, 2014; Liu et al., 2022). When describing the qualities of the ideal dance partnership, an elite dancer suggested that a partner who can help them improve their dancing skills and obtain excellent competitive performance is perfect (Marion, 2006). Second, however, in Asia like China, there is a strong emphasis on obligation between DanceSport couples (Liu et al., 2022). The obligation is ruled by “*renqing (favor)* or *mianzi (face)*” based on Confucianism, which is based on personal feelings rather than commercial law to safeguard obligations. When people fail to comply with the rules, it will lead to public criticism. To avoid such criticism, Chinese people present a “public-self” to others to preserve their face or the face of others, while maintaining their true and selfish “private self” (Chang, 2012). Although Westerners are accustomed to the two concepts of losing and saving face, using and understanding these metaphors, the Chinese do it better; they are also very good at giving face, taking care of the face, and fighting for the face to maintain a harmonious relationship (Hwang, 1987; Peng, 1998; Seligman, 1999).

**Expressive ties** (ET) refer to the emotional bond containing instant intimacy produced by elite dancer couples during the full process of taking DanceSport competitions as the goal. Expressive ties involve both instant intimacy (Ericksen, 2011, pp. 20–21) and prolonged emotion (Brewiñska and Poczwardowski, 2011; Yang, 2015). To be more specific, on the one hand, when dancers dance together for the pleasure of communication, an instant intimate relationship will be formed between them. For example, the dancers will demonstrate the romance and passion between the sexes in Rumba (known as the “soul of Latin dance” and the dance of love). Meneau (2020), a scholar at the University of Salzburg, Austria who specialized in gender and DanceSport research, even described the Rumba performance of the world’s top competitors Gabriele Goffredo and Anna Matus in the 2014 Brno Open: “*He approaches her from behind until his chest touches her back, then thrusts his hands to her lower thighs and caresses her upwards. After grabbing her waist, he pushes her away and sharply pulls her back to him, provoking an impact of her back against his ribcage.*” On the other hand, a partnership between DanceSport couples contains factors such as care and appreciation. To continuously improve professional skills and obtain excellent competitive performance, dyadic couples not only need to spend a lot of time and energy in training but also need to travel to different cities and countries to participate in competitions. The daily life, learning, and training of the two are highly intertwined. Therefore, there is a shared interactive memory system (Transactive Memory System, TMS) between the dance partners to jointly encode, store, and retrieve information (Grysman et al., 2020). According to Wegner (1987), dance partners will have an intense sense of identity and emotional bonds during the process of cooperation. In the case of elite dancers, the interaction between them exists in both professional training and private life. Dance partners are not only best friends but also life partners (Brewiñska and Poczwardowski, 2011). In fact, ballroom dance is about love and also about sex (Ericksen, 2011, p. xii), as Brewiñska and Poczwardowski (2011, pp. 27) mentioned that rapport between dance partners was essential whether driven by romantic attraction to each other or simply by a shared passion for the dance. This is mainly because the technical acquisition of DanceSport requires long-term cooperation and training. During this period, the partners are always together, and the connection between the two is intimacy, passion, and immersion (Yang, 2015). They must exhibit passion and emotion during dancing. Very often, high-class dancing couples are also pairs in life (Majoross et al., 2008; Brewiñska and Poczwardowski, 2011).

**Interpersonal perception** (IP) refers to the ability of elite dancer couples to share and expose each other during the whole process of taking competition as the goal so as to sensitively perceive the psychological and behavioral tendencies of dance partners, including revealing their hearts, sharing, and understanding each other. As depicted by Dan (2012), interpersonal abilities are the engine of artistic communication and the wish to dance comes from the wish to communicate and to feel a connection with the partners. Partners show themselves to each other, get to know each other, and create a heart-to-heart awareness of each other, which is so vital that Myung et al. (2010) defined the dimension of “*partner harmony*” in the partnership between partners after interviewing registered Korean elite dancers and set up the item “understand the personality of the partner” in the dance partnership questionnaire. However, in the Chinese context, it may be more important to understand the dance partner. More specifically, for the Chinese, intimacy is the result of the interaction between individuals as boundaries melt away, i.e., two people violate each other and then reach a state of “you and I are indistinguishable” (Jamieson, 1998; Friedman, 2005). The dance couples are more likely to raise the training issues into love issues, for the expressive ties between the partners will always break the regular operation mode of professional cooperation, which can lead to quarrels and conflicts. Therefore, the elite dancers need to understand their partner through communication, share their inner thoughts, and create a heart-to-heart connection, which can facilitate the resolution of arguments and differences brought about by the blurred role boundaries.

### 1.3. The aim of the present study

Although research on the athlete–athlete partnership framework is an important research direction, the research is still in its infancy. According to Poczwardowski et al. (2019), the most important work of researchers is to build a complete and ambitious system out of a large theoretical wasteland, focus on the screening and dimensional constructions of two-person interpersonal relationships (i.e., analytical framework construction), and then incorporate it into a theoretical model of how two-person interpersonal relationships arise in relation to other variables (Poczwardowski et al., 2019). As the DanceSport partnership research is in its primary stage of theoretical research, literature on the partnership between DanceSport couples is sparse (Majoross et al., 2008; Liu et al., 2022). Therefore, the purpose of our study was to develop a scale to measure the partnership between DanceSport couples—Partnership Scale-DanceSport Couples (PS-DSC). The following four studies were conducted. The first study aimed to generate initial items and refine them. The second study was to examine the factor structure of the PS-DSC items identified in study 1 through exploratory factor analyses. The third study was to further examine the factor structure of the PS-DSC items using confirmatory analyses. The fourth study provided an assessment of composite reliability, discriminant validity, and convergent validity.

## 2. Study 1: Initial development of the scale

Study 1 was to generate items that captured the three partnership dimensions between DanceSport couples and to evaluate and refine the initial PS-DSC item pool.

### 2.1. Expert interviews to validate definitions

The operational definition and the three-dimensional structure (instrumental ties, expressive ties, interpersonal perception) of the partnership between the DanceSport couples were validated using expert interviews and were finally approved by one researcher from the Institute of Psychology, Chinese Academy of Social Sciences, one Associate Researcher from the Institute of Psychology, Chinese Academy of Sciences, three masters from the School of Psychology at Beijing Normal University, and 6 DanceSport experts from sports colleges and universities (The Beijing University of Physical Education, The Capital Institute of Physical Education, The Wuhan Institute of Physical Education, and The Xi’an Institute of Physical Education; by telephone and field visits).

### 2.2. Elite dancer interviews

The initial items were generated through 20 excellent Chinese DanceSport elite dancers (Table 1) who have a training period of more than 7–20 years, more than 3 years of working experience with a fixed partner, and exhibited superior performance in competitions. This sample was confirmed in advance with two competitors who had won championships and 4 DanceSport experts from Beijing Sports University and Xi’an Sports University. The sample consisted of 8 men and 12 women. Their average age was 24.30 years (range = 19–31, SD = 3.80). On average, they have been dancing for 13.85 years (SD = 4.44) and experienced 4.95 years of partner time (SD = 2.82). The interviewees almost covered the excellent dancers of each group class that can represent the level of Chinese sports dancers. Therefore, the results obtained through the interviews were undoubtedly credible and representative of understanding and generalizing the items of PS-DSC.

**TABLE 1:** Basic information of the interviewed elite dancers.

Discipline	Code	Sex	Age (year)	Training time (year)	Partner time (year)	Domestic best results	Length of interview (minute)	Interview recordings (words)
B	A	M	31	20	14	First (PRO)	65	11935
B	B	F	25	12	6	Third (PRO)	45	6297
B	C	M	26	16	4	Forth (PRO)	43	4376
B	D	M	23	13	3	Forth (PRO)	39	1863
B	E	F	29	21	3	Sixth (PRO)	27	4693
B	F	F	25	18	6	Sixth (PRO)	34	4862
B	G	F	32	20	5	Second (PRO-RS)	26	2717
B	H	M	26	20	7	Third (A-group)	67	9453
B	I	F	27	16	3	Third (A-group)	39	4018
B	J	M	20	15	8	Third (A-group)	36	4937
B	K	M	24	8	3	Forth [A(RS)-group]	21	2332
L	L	M	28	10	7	Second (PRO)	50	8699
L	M	F	25	15	3	Third (PRO)	50	8500
L	N	F	22	10	3	First [A(RS)-group]	53	11012
L	O	F	21	10	8	Third (A-group)	26	3399
L	P	F	22	13	4	Eighth (A-group)	30	4219
L	Q	F	19	10	3	Sixth [A(RS)-18 years-group]	32	3521
L	R	M	19	7	3	Sixth [A(RS)-18 years-group]	43	8026
L	S	F	21	8	3	First [A(RS)-group]	43	4468
L	T	F	21	15	3	First [A(RS)-group]	27	6532

*B, ballroom dance; L, Latin American Dance; PRO, professional; PRO-RS, professional rising star, A(RS)-group, A-rising star.

The total length of the interviews was 796 min. The interview period spanned 45 days from 29 May 2019 to 12 July 2019. The reason for the long span was due to the intensive domestic and international events as most elite dancers participated in many competitions and it was hard to make appointments with them to get the interview. Finally, the transcription of the voice material into text material was 115,869 words.

### 2.3. Initial item generation and item refinement

By analyzing the results considering the Korean DanceSport partnership scale (Lee and Dawes, 2005; Myung et al., 2010; Kim et al., 2011) and the recommendations of DeVellis (2011), we generated 35 items using the definitions and key characteristics of each component. The following 20 items were chosen by 10 experts (Table 2) for their clarity, readability, relevance, similarity to other items generated, items in existing scales, and the degree to which they adhered to the specified criteria.

**TABLE 2:** Identification experts list (*n* = 10).

Code	Title	Specialty
1	Professor	Sport psychology
2	Professor	Social psychology
3	Professor	Psychometrics
4	Professor	Culture psychology
5	Professor	The teaching and training of DS
6	Doctor	The teaching and training of DS
7	Doctor	The teaching and training of DS
8	Doctor	The teaching and training of DS
9	Champion	Champion of Latin dance competition
10	Champion	Champion of ballroom dance competition

DS, DanceSport.

## 3. Study 2: Scale refinement and EFA

The results generated by study 1 awaited empirical support (or invalidation) regarding the partnership between DanceSport couples. In study 2, we explored the factor structure and the psychometric properties of the second revised pool of twenty items identified in study 1 through EFA, which was considered a useful method of data reduction when developing or refining a scale (Anderson and Gerbing, 1988) and to ensure the refinement of each subscale before proceeding to CFA analysis.

### 3.1. Participants

The sample reserved for study 2 consisted of 289 young elite dancers (134 men and 154 women). The average age of the elite dancers was 20.96 years (range = 14–33, SD = 3.40). On average, they had been dancing for 6.98 years (SD = 4.12), had dedicated 9.28 h (SD = 9.65) to training and competition per week, and had experienced 21.26 months of partner time (SD = 21.57).

### 3.2. Data analysis

The purpose of data analysis was to further optimize the items of the scale and assess the factor structure of the subscales. Before conducting the EFA, the following information was collated and used to decide which items to retain: (a) critical value, item-total correlation, Cronbach’s alpha after the item is deleted, commonality, and factor loading and (b) the value of the KMO (Kaiser–Meyer–Olkin) and Bartlett tests, and an oblique rotation that was investigated to identify the factor structure (Anderson and Gerbing, 1988).

Then, EFA was calculated using IBM SPSS Statistics 22. We wanted an empirical summary of the dataset by principal components analysis (Tabachnick and Fidell, 2007) and decided that the number of factors were evident through an unrotated factor solution. Then, Kaiser’s criterion (Kaiser, 1960) and the scree plot (Cattell, 1966) were adopted to determine the number of factors in each subscale.

### 3.3. Discussion

Preliminary tests before EFA revealed that all items met the criteria (critical value ≥3.50, item total correlation ≥0.40, Cronbach’s alpha after the item was deleted did not get smaller, communality ≥0.200, and factor loading ≥0.45), except for the item “X15.” The reason was that Cronbach’s alpha of this scale remained 0.945 when it was deleted (see Table 3). In addition, the value of the KMO (Kaiser–Meyer–Olkin) and Bartlett tests was suitable for factor analysis (0.926, *p* < 0.001).

**TABLE 3:** Summary of DanceSport partnership scale.

Item	Critical value	Item total correlation	Cronbach’s Alpha after the item is deleted	Communality	Factor loading	Result
X1	10.286	0.634**	0.944	0.518	0.598	Retain
X2	12.554	0.709**	0.942	0.803	0.853	Retain
X3	10.698	0.631**	0.943	0.705	0.787	Retain
X4	14.236	0.790**	0.941	0.721	0.716	Retain
X5	15.396	0.804**	0.940	0.735	0.759	Retain
X6	9.346	0.619**	0.944	0.831	0.872	Retain
X7	12.953	0.682**	0.943	0.726	0.802	Retain
X8	11.801	0.658**	0.943	0.530	0.679	Retain
X9	12.718	0.753**	0.941	0.714	0.792	Retain
X10	10.852	0.670**	0.943	0.790	0.811	Retain
X11	13.021	0.719**	0.942	0.834	0.871	Retain
X12	12.95	0.731**	0.942	0.728	0.505	Retain
X13	10.141	0.660**	0.943	0.512	0.606	Retain
X14	12.747	0.725**	0.942	0.848	0.878	Retain
X15	8.916	0.490**	0.945	0.500	0.618	Delete
X16	14.584	0.806**	0.940	0.722	0.731	Retain
X17	14.813	0.807**	0.940	0.737	0.712	Retain
X18	15.593	0.792**	0.941	0.707	0.714	Retain
X19	12.857	0.724**	0.942	0.668	0.766	Retain
X20	9.522	0.563**	0.944	0.554	0.707	Retain

Cronbach’s alpha of this scale is 0.945. **means *P* < 0.01.

**FIGURE 1:**
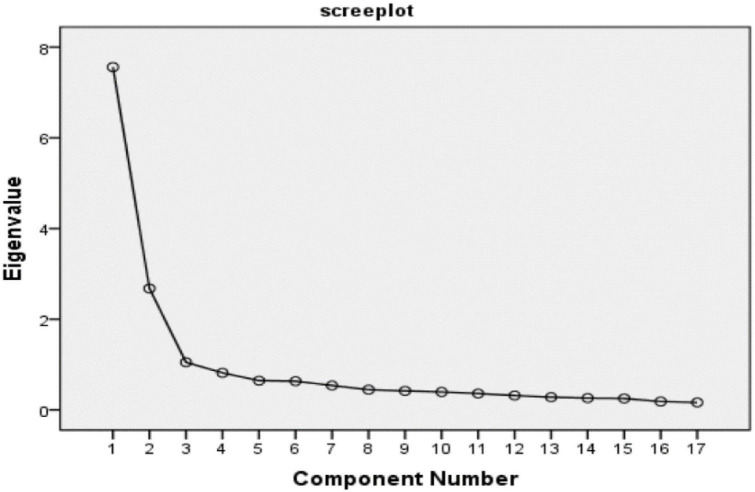
Scree plot of the DanceSport partnership.

The outcome of the item analysis turned out to be the suitability of the data for EFA which aimed to recombine disordered variables and explore the potential structural relationship between the variables (Lai, 2014). This study conducted EFA on the remaining nineteen items. Using principal component analysis, three factors were obtained according to Kaiser’s criterion, the eigenvalues of which were greater than 1. It can be seen from the scree plot that, after the third factor, the slope becomes gentle and the factor starting from the fourth factor presents a straight line (see Figure 1), indicating that the contribution of these factors to the original variables can be almost ignored. These two tests proved that it was appropriate to extract the three factors. In addition, an oblique rotation was performed to identify the factor structure (Anderson and Gerbing, 1988), and the results are shown in Table 4.

**TABLE 4:** The summary of factor analysis of Partnership Scale-DanceSport Couples (PS-DSC) (*N* = 289).

Items	Factor loading			Communality
	Factor 1	Factor 2	Factor 3	
X14 cooperate with my partner will get closer to my goal	0.889			0.874
X11 cooperate with my partner will make me grow professionally	0.876			0.842
X2 cooperate with my partner will achieve my goal	0.866			0.820
X7 cooperate with my partner promote my personal ability	0.813			0.733
X3 my partnership is mutually beneficial and win-win	0.809			0.709
X20 I obligately follow the training plan agreed with my partner	0.680			0.503
X13 I and my partner should support each other	0.598			0.502
X9 In dance training or competition, my partner often gives me a fresh feeling		0.816		0.746
X19 I find my partner very attractive		0.780		0.685
X4 In dance training or competition, I and my partner are full of passion		0.769		0.732
X5 I and my partner understand each other		0.747		0.730
X16 I get along with my partner		0.730		0.726
X18 I and my partner appreciate each other		0.703		0.707
X17 I and my partner care about each other		0.695		0.736
X8 I feel relaxed and happy with my partner without pressure		0.663		0.522
X6 I feel I really know my partner			0.877	0.830
X10 I feel my partner really knows me			0.806	0.775
X12 I and my partner are tied together			0.707	0.774
X1 I am willing to share my true thoughts with my partner			0.655	0.561
eigenvalue (unrotated)	9.68	2.61	1.19	
variance explained (%)	27.44	26.61	16.89	
the amount of variance explained (%)	27.44	54.05	70.94	

According to Table 4 and Figure 1, the results of the three factors were explained as follows:

The items “X14, X11, X2, X7, and X3” in factor 1 showed that dance partnership was regarded as a means to obtain competitiveness and performance, which was in line with the “economic man” hypothesis. The items “X20: I obligately follow the training plan agreed with my partner” and “X13: I and my partner should support each other” highlighted the sense of obligation to hide utilitarianism, which indicated that the couples need to undertake certain obligations to obtain excellent competitive performance. Chinese dance partners follow the law of “*renqing*” and “*mianzi*” which emphasized the role of the obligation of desalinating utilitarian purposes so as to avoid the embarrassment caused by even exchange, which was different from the contractual obligation corresponding to power in the West. To sum up, the stickiness between the utilitarian tendency and the sense of obligation was strong. Based on the above seven items, factor 1 was named the obligatory instrumental tie.

The items “X5, X16, X18, X17, and X8” in factor 2 revealed the long-term accumulated emotions between dance partners. This was in line with the requirements of the “social man” hypothesis, that is, the emotional needs were the basic needs of people. Competitive sports events, including DanceSport, require long-term training to acquire the skill (Reynolds, 1998). Some participants in our study were between the ages of 17 and 28 years. They have higher emotional needs and greater emotional fluctuations. In addition, the three items “X9, X19, and X4” reflect a short-term emotional state full of desire formed by the contestant and his partner in the field. In addition, expressive tie was included in Chinese ‘Guanxi’ (Kipnis, 1997). Based on the above eight items, factor 2 was named expressive tie.

The items “X6 and X10” in factor 3 reflect the elite dancers’ requirements for perceiving all aspects of their partners. Item “X12” reflected that the elite dancers wanted to express a tacit understanding with their partners in dance and life. Item “X1” reflected the elite dancers’ need for self-disclosure, under the reciprocal effect of disclosure, and individual self-disclosure, which will lead to the disclosure of the others (Miller, 1990) and ensure full communication between dance partners in training and avoided conflicts as in the cooperative context (Tjosvold, 1981). These items described the desires of elite dancers to perceive the ideas and attitudes of their partners and to understand their views of themselves. As the dancers will have cognitive differences or even conflicts due to individual differences, they need to constantly strengthen their interaction and communication with the perceptual object, timely adjust their state based on their cognitive ability and attitude toward their partners, and ensure the development and maintenance of the partnership. In addition, the stability of interpersonal relationship depends on the formation of certain relationship perception (Baumeister and Leary, 1995). Combining the above four items, factor 3 was named interpersonal perception.

## 4. Study 3: Scale confirmation and CFA

The purpose of study 3 was to further examine the three-factor 20-item structure model that is identified in study 2 using confirmatory factor analysis (CFA) through SEM techniques.

### 4.1. Participants

The sample reserved for study 3 consisted of 288 elite dancers (115 men; 172 women). The average age of the elite dancers was 19.30 years (range = 14–31, SD = 3.30). On average, the elite dancers have been dancing for 7.70 years (SD = 4.40), dedicating 9.67 h (SD = 4.17) to training and competition per week and experiencing 18.65 months of partner time (SD = 23.19).

### 4.2. Data analysis

Confirmatory factor analysis examined whether the relationship between a factor and the corresponding measurement term conforms to the researchers’ theoretical assumptions, we used SEM to verify the fitting degree of the proposed model to the actual observation data obtained from EFA. The SEM mode analysis adopted the estimation method of complete information technology, which was built on the bases of the normal theory. Therefore, the selection of the estimation method was affected by the nature of the sample distribution, and the data of this study needed to be conformed to the multivariate normal distribution. The normality test required that the absolute values of the skewness coefficient and kurtosis coefficient were less than 1.96. The results of this study showed that (as shown in Table 5) the skewness value of the observed variables of DanceSport partnership was between −1.444 and 0.084 and that the kurtosis value is between −0.022 and 2.865, and hence, the data of our study tend to be normally distributed.

**TABLE 5:** Descriptive statistical summary table of observed variables for the DanceSport partnership questionnaire (*N* = 288).

	Min	Max	M	S	Skewness	Kurtosis
X1 I am willing to share my true thoughts with my partner	1	5	3.86	1.006	−0.669	0.006
X2 cooperate with my partner will achieve my goal	1	5	4.04	0.923	−1.039	1.272
X3 my partnership is mutually beneficial and win-win	1	5	4.17	0.92	−1.319	1.863
X4 In dance training or competition, I and my partner are full of passion	1	5	3.62	0.922	−0.593	0.375
X5 I and my partner understand each other	1	5	3.60	0.979	−0.565	0.04
X6 I feel I really know my partner	1	5	3.17	1.073	−0.208	−0.279
X7 cooperate with my partner promote my personal ability	1	5	4.18	0.859	−1.112	1.504
X8 I feel relaxed and happy with my partner without pressure	1	5	3.56	1.017	−0.511	−0.038
X9 In dance training or competition, my partner often gives me a fresh feeling	1	5	3.3	0.952	−0.181	−0.188
X10 I feel my partner really knows me	1	5	2.99	1.088	−0.084	−0.347
X11 cooperate with my partner will make me grow professionally	1	5	4.27	0.815	−1.229	1.966
X12 I and my partner are tied together	1	5	3.31	1.087	−0.385	−0.223
X13 I and my partner should support each other	1	5	4.35	0.787	−1.444	2.865
X14 cooperate with my partner will get closer to my goal	1	5	4.16	0.867	−1.179	1.821
X16 I get along with my partner	1	5	3.7	0.938	−0.764	0.624
X17 I and my partner care about each other	1	5	3.56	1.051	−0.583	−0.022
X18 I and my partner appreciate each other	1	5	3.35	1.029	−0.482	−0.039
X19 I find my partner very attractive	1	5	3.31	1.091	−0.395	−0.37
X20 I obligately follow the training plan agreed with my partner	1	5	4.13	0.816	−1.1	1.997

We used CFA with maximum likelihood estimation to examine whether the hypothesized three-factor structure was supported by the data. Multiple goodness-of-fit measures were used to indicate the model fit [*χ^2^/df* < 3 (Kline, 2005); GFI, AGFI, CFI, NFI ≥ 0.90; PNFI, PGFI < 0.8 (Kline, 1998)]. The value of RMSEA was between 0.05 and 0.08. When the value was below 0.05, it indicated that the model fitting degree was incredibly good (Browne and Cudeck, 1992). The results of this study (Table 6) showed that the model fitting index was not ideal. According to the standard of a minimum of 3 items in each dimension, this model still has a lot of room for correction, and hence, the model would be revised and retested.

**TABLE 6:** Initial model fitness result of DanceSport partnership.

	χ*^2^*	df	χ^2^/df	GFI	AGFI	NFI	CFI	PNFI	PGFI	RMSEA
Initial Model	568.686	149	3.817	0.818	0.768	0.868	0.899	0.757	0.641	0.099

In this study, the items were deleted according to the M.I. modification index, meaning that, between two items with higher M.I. indices, the item with the highest M.I. value than other items was first selected for deletion to eliminate the serious collinearity problem between items. In this study, a total of 7 model revisions were carried out and 7 items were deleted in turn as shown in Table 7. Table 8 shows the model fitting indicators of the 7 revisions. The results showed that, after the 7th revision, the model fitting indicators of the model met the strict requirements of the psychometric test.

**TABLE 7:** Items deleted after revised.

Revise	Deleted items
1	X5 I and my partner understand each other
2	X10 I feel my partner really knows me
3	X13 I and my partner should support each other
4	X9 In dance training or competition, my partner often gives me a fresh feeling
5	X8 I feel relaxed and happy with my partner without pressure
6	X19 I find my partner very attractive
7	X7 cooperate with my partner promote my personal ability

**TABLE 8:** Results of the confirmatory factor analysis for DanceSport Partnership (*N* = 288).

	χ^2^	df	χ^2^/df	GFI	AGFI	NFI	CFI	PNFI	PGFI	RMSEA
1	483.418	132	3.662	0.841	0.794	0.877	0.907	0.757	0.649	0.096
2	385.642	116	3.324	0.861	0.817	0.890	0.920	0.759	0.653	0.090
3	317.014	101	3.139	0.875	0.832	0.904	0.932	0.761	0.650	0.086
4	237.892	87	2.734	0.898	0.860	0.921	0.948	0.763	0.651	0.078
5	169.581	74	2.292	0.923	0.891	0.940	0.965	0.764	0.651	0.067
6	141.954	62	2.290	0.930	0.898	0.945	0.968	0.751	0.634	0.067
7	119.592	51	2.145	0.938	0.905	0.957	0.975	0.739	0.613	0.068

Then, the second-order model test was conducted, and the results showed that χ^2^/*df* = 2.319, *RMSEA* = 0.068, *SRMR* = 0.037, *GFI* = 0.938, AGFI = 0.906, *NFI* = 0.957, *CFI* = 0.975, *PNFI* = 0.740, and *PGFI* = 0.614. This reveals that the fitting effect was good.

## 5. Study 4: Reliability and validity test

### 5.1. Participants

In this study, the event held by the Chinese DanceSport Federation (Beijing Station) was selected as the carrier to obtain the results of the A(RS)-group and A-(RS) group elite dancers (after experts’ identification, the elite dancers in these two groups had a deep understanding of the partnership). The sample consisted of 242 young elite dancers, of which 122 were men and 120 were women. Of them, 91 have been dancing for 0–5 years, 107 for 5.1–10 years, 28 for 10.1–15 years, and 16 for over 15 years. A total of 151 elite dancers spend 0–12 months with their partners, 58 for 13–36 months, 22 for 37–60 months, and 11 for over 60 months.

#### 5.1.1. Reliability analysis

A composite reliability of >0.6 was one of the criteria for judging the intrinsic quality of the model. The average variance of >0.50 indicated that the intrinsic quality of the model was ideal (Bagozzi and Youjae, 1988; Hair et al., 2009). Using SEM to calculate the reliability of the individual topics and potential variables, the results are shown in Table 9. The results showed that the internal structural fitness of the DanceSport partnership model developed in this study was in line with the ideal level and has good internal quality.

**TABLE 9:** Component reliability of latent variables and average variation extraction of Partnership Scale-DanceSport Couples (PS-DSC).

Latent variable Observable variable	Estimate	CR	AVE
**Obligatory instrumental ties**		0.93	0.72
X14 cooperate with my partner will get closer to my goal	0.883		
X11 cooperate with my partner will make me grow professionally	0.932		
X2 cooperate with my partner will achieve my goal	0.896		
X7 cooperate with my partner promote my personal ability	0.851		
X20 I obligately follow the training plan agreed with my partner	0.667		
**Expressive ties**		0.87	0.65
X4 In dance training or competition, I and my partner are full of passion	0.488		
X16 I get along with my partner	0.886		
X18 I and my partner appreciate each other	0.871		
X17 I and my partner care about each other	0.893		
**Interpersonal perception**		0.72	0.89
X6 I feel I really know my partner	0.854		
X12 I and my partner are tied together	0.832		
X1 I am willing to share my true thoughts with my partner	0.863		

#### 5.1.2. Validity test

##### 5.1.2.1. Discriminant validity

Discriminant validity refers to the low correlation or significant difference between the dimensions and the potential traits represented by dimensions. This study used the correlation between the dimensions and the correlation between dimensions and total score to judge the differential validity of the scale. The Pearson correlation analysis showed that the correlation coefficients between each dimension of DanceSport partnership, namely, the subscale and the total score, were 0.838–0.869, which reached a significant level (*P* < 0.05). The correlation coefficient between dimensions is 0.555–0.631 and the scale has good differential validity.

##### 5.1.2.2. Convergent validity

Convergent validity refers to the items that measuring the same potential traits will fall on the same factor dimension, and there was a high correlation between the items and the measured values between tests. Only when the factor load of each observed variable on its latent variable was not less than 0.45, the observed variable was sufficient to reflect its constructed potential variable (Bentler and Wu, 1993). In this study, the dimensional factor loads of obligatory instrumental ties, expressive ties, and interpersonal perception were all moderately and highly significant and exceed the threshold of 0.45. Therefore, PS-DSC has good convergent validity.

## 6. General discussion

This study aimed to develop a scale to measure partnership between DanceSport couples. The four studies outlined highlight the key stages in the initial development and validation of the PS-DSC. In stage 1, outlined in study 1, PS-DSC items were generated and refined through literature analysis, interviews with players with rich partner experience, and expert identification. In stage 2, outlined in studies 2 and 3, exploratory and confirmatory analyses were used to further examine the structure of PS-DSC items. In stage 3, outlined in study 4, composite reliability, discriminant validity, and convergent validity were assessed. The result of this process was a three-factor 13-item scale.

The development of the PS-DSC was underpinned and guided by some considerations. First, we considered the partnership between DanceSport couples to be composed of three distinct and interrelated components representing obligatory instrumental ties, expressive ties, and interpersonal perception. We found strong support for this hypothesized three-factor structure across measurement models and samples. Second, we found that the 3 dimensions did work on measuring the partnership between DanceSport couples using practical and theoretical analysis. For dancers, they can choose partners who share the same goals as them by scoring using the scale. For example, elite dancers who tend to have expressive ties can choose partners with higher scores in expressive ties and interpersonal perception. The coaches can also help the elite dancers match their partners by scoring on the scale. This enables the dance partners to stay together for a long time, cultivate more tacit skills of pairs, provide a good interpersonal environment for elite dancers to break through more excellent competitive performance, and make certain contributions to the world dance sport competition.

### 6.1. Obligatory instrumental ties

This dimension integrates both instrumental ties and obligatory ties (Liu et al., 2022). On the one hand, it is in line with the reciprocal endeavor dimensions of the partnership between Korean DanceSport couples (Myung et al., 2010), the commitment dimension in the 5C theory of athlete–athlete partnership (Poczwardowski et al., 2019), and the 3C theory of the coach–athlete relationship (Jowett and Meek, 2000) in terms of instrumental elements that emphasized exchange and utilitarianism. Because DanceSport partners are a community sharing common goals and tasks (Vegt et al., 2010), highly interdependent dance dyads emphasize the exchange of “gain” and “give” results with the goal of obtaining competition rankings. Therefore, in our study, items “X7 cooperate with my partner promote my personal ability,” “X11 cooperate with my partner will make me grow professionally,” “X12 I and my partner are tied together,” and “X14 cooperate with my partner will get closer to my goal” all showed good reliability and validity in statistics when tested.

However, for relationship in China was particularistic (Farh et al., 1998) on the other hand, we emphasized the sense of obligation between DanceSport couples. As Alan Fiske (1992) pointed out, obligations can only be discussed in different relationships, which meant that the sense of obligation has specific cultural characteristics. For example, in the items of the DanceSport partnership scale developed by Korean couples, the items: “my partner and I will be full of vitality when dancing in front of the crowd” and “my partner and I have left a deep impression on the audience” are considered to be prejudiced against Korean male groups (Yang, 2015). As Hwang (1987) states, the component of obligation in partnership between Chinese dance couples was a mixture of expressive and instrumental ties, following the “*renqing*” rule, as opposed to the “contractual obligations” that emphasized the role and obligation of weakening utilitarianism. Therefore, in our study, the item “X20 I obligately follow the training plan agreed with my partner” was indispensable for elite dancers. For example, one elite dancer [A(RS)-group] in China stressed that “I feel guilty to see my partner practicing dancing in the glass room.” In addition, one professional elite dancer mentioned that “we are not a couple, we are fighting together for a common goal, but I still hope not to look too much utilitarian, we are all friends.” Therefore, we believed that obligatory instrumental ties were an important and special dimension of DanceSport partnership in the Chinese context.

### 6.2. Expressive ties

This dimension contains intimacy caused by long-term contact and short-term passion. It is in line with the partner care dimension of partnership (Myung et al., 2010), the closeness dimension in the 5C theory of athlete–athlete partnership (Poczwardowski et al., 2019), and the 3C theory of the coach–athlete relationship (Jowett and Meek, 2000) in terms of long-term intimacy. Based on Robert Stemberg’s ternary theory of love (Sternberg and Grajek, 1984), scholars believed that intimacy included the characteristics of mutual understanding, caring, and support, which was highly positively correlated with being understood and appreciated (Fitzsimons and Kay, 2004). As for DanceSport elite dancers, they needed to increase the frequency of interdependence in the process of long-term training and competition to establish close contact with their dance partners to learn the technique of double cooperation together (Ericksen, 2011). Therefore, romantic relationships, marriage relationships, or similar kinship relationships will be derived from the interaction of partners. For example, in the interview with this study, one elite dancer, the third (PRO) in China mentioned that “in the first 5 or 6 years when we left hometown and danced together in Shanghai, we only had each other and took care of each other. We were like family.” Therefore, we proposed items “X16 I get along with my partner,” “X17 I and my partner care about each other” and “X18 I and my partner appreciate each other,” which can measure the intimacy and depth with their dance partner.

However, the short-term passion, which is very vital to competitive performance, was seldom proposed in studies of a partnership between elite dancers′ dyads, for the athlete–athlete partnership in different sports presented distinctive characteristics (Poczwardowski et al., 2006). DanceSport is based on romantic fantasy (Harman, 2019) which openly expresses emotions, shows the body between dance partners, and highlights the intimate relationship between the two sexes (Ericksen, 2011). The intimate body movement makes the dancers sink into the love world full of male and female under the action of a mirror neural mechanism. Peters (1992), a Yale University teacher who has won American DanceSport competitions, wondered about the lust between dance partners. As she said, driven by passion, dancers’ lives revolved around dancing, with enthusiasm and dedication, as they practiced complex tricks repeatedly. Whether on or off the dance floor, they were swallowed up by their own imagination of dance, such as practicing courtship rituals or erotic love in the tango so that people can see fascinating and attractive relationships immediately. She also wondered if they were still dance partners off the dance floor.

So, the results of this study showed that the item “X4 In dance training or competition, I and my partner are full of passion” has good statistical significance for the formation of the scale.

### 6.3. Interpersonal perception

Comparing the dimensions and scales of partnership between DanceSport couples (Myung et al., 2010; Kim et al., 2011), the interpersonal perception was a separate dimension in partnership between DanceSport couples in our study. Previous literature includes the components of interpersonal perception, such as “understand personality” (Myung et al., 2010). However, we believed that only one item cannot reflect the importance of interpersonal perception. The “Window theory” proposed by American psychologists Luft and Ingham (1955) stressed that with the development of interpersonal relations and the gradual establishment of intimacy, the “territory” of the open self, driven by the two interactive processes of self-disclosure and feedback from others, continued to expand outward along the two dimensions of depth and breadth so that the “territory” of the blind self, the hidden self, and the unknown self were constantly compressed and the individual’s understanding of himself was more and more comprehensive and profound. Therefore, the more clearly one can disclose their internal thoughts, attitudes, emotions, likes and dislikes, and expertise, among others to others, the better others can understand and know themselves. DanceSport provides a window for students to happily listen to and understand each other through body nonverbal communication (Ogilvie, 2017; Pisu et al., 2017).

Overall, the importance of interpersonal perception is reflected in its ability to better maintain and develop a partnership between DanceSport couples. Since, based on the field theory, the psychological connection between the DanceSport partners was a combination of instrumental ties and expressive ties influenced by different laws of operation, where the instrumental ties occur in the training competition arena, the expressive ties occur in daily life, and the dancers are likely to turn the cooperation issue into a love issue, making the intimate partnership between the dyads in practice break the conventional mode of operation of professional cooperation (Liu et al., 2022). In the absence of contractual constraints, the maintenance and development of partnerships will face challenges (Wang, 2018). In addition, based on the cultural psychology perspective, Markus and Kitayama (1991) put forward the self-concept analysis framework of “*independent self*” and “*interdependent self*” based on the characteristics of cultural differences, which emphasized that Asian people were more inclined to find themselves by relying on others. This was an inclusive individualistic interpersonal relationship, in which the individual’s self-boundary was rheological and the establishment of expressive ties would accelerate the blurring of this boundary (Sampson, 1988). The expressive ties are the result of interaction after the boundary between individuals melts in China. The two people invade each other and then reach the state of “no distinction between you and me” (Jamieson, 1998; Friedman, 2005). Thus, elite dancers are likely to raise cooperation issues to love issues, to make the intimate relationship break the mode of professional cooperation in practice. In the case of only “*renqing*” without contractual constraints, the maintenance and development of partnerships will face challenges. Therefore, our study came up with three items- “X1 I am willing to share my true thoughts with my partner,” “X6 I feel I really know my partner,” and “X12 I and my partner are tied together” to measure whether couples can understand their partners’ dance style, technical characteristics, and possible problems in dance (Pistole, 2003), without just caring about “understanding personality” between dance partners (Myung et al., 2010). This interactive function between partners makes dance movements be used as an intervention means to help participants interact with each other (Pisu et al., 2017).

## 7. Strength and limitations

The present study demonstrates some strengths. It provides a scale to evaluate the quality of the partnership between DanceSport couples and promote the development of DanceSport research in depth. Creating a scale of DanceSport partnerships will be beneficial for sports dancers and coaches. In addition, as a future study, we believe it will be good to recruit non-Chinese experiment participants and prove it. Another strength of the present study is that our study conforms to the development direction of sports psychology. Poczwardowski, Barott, and Jowett have stressed that research about partnerships among dyadic sports can be considered an important topic in 2006. After 12 years, in the theme (Sport-a path to peace between people)” under the 4th International Scientific and Practical Conference, Russian scholar Maximova (2018) reviewed the development of synchronized swimming in recent years with the opening of a dyadic sport, stressing once again that partnership has a tendency to develop into a new discipline in sport and raise expectations for the construction of equal and successful partnerships. In addition, a growing trend in the transformation of sports culture was to organize double event competitions, such as beach volleyball, figure skating, and ballroom dancing (Moskatova and Maltsev, 2018), and the peers seem to be important at all career stages (Kram and Isabella, 1985). Therefore, the study paid more attention to the partnership. The concept of partnership has appeared in the field of sports psychology but has not received much attention in DanceSport so far. Our research systematically discussed the partnership between DanceSport couples through qualitative and quantitative research.

This study has some limitations. Since we are using self-reported forms to obtain information, the results may be influenced by social expectations. In addition, we are not sure whether the results of this study can be extrapolated to other dyadic sports. The reason is that unique connotations to athlete–athlete partnership were influenced by the style of sports characteristics and hence there may be differences in athlete–athlete partnership components (Gaudreau et al., 2010; Poczwardowski et al., 2019). Therefore, future research should explore the analysis framework of the athlete–athlete partnership in different events to advance the theory of partnership in different sports and enrich the theory of competitive performance (Wickwire et al., 2004), which provides challenges and opportunities for discovering new concepts and theories in the partnership of different dyadic sports events.

## 8. Conclusion

Our research was to apply the construct of a partnership between DanceSport couples and develop the scale to measure it. Here, we have reported on key stages involved in the development and initial validation of this new scale. Based on these analyses, the PS-DSC shows evidence of good factorial validity and reliability.

## Data availability statement

The raw data supporting the conclusions of this article will be made available by the authors, without undue reservation.

## Ethics statement

The present study was conducted in accordance with the Declaration of Helsinki and was also approved by the Ethics Committee of the Wuhan Sport University. Written informed consent was obtained from all subjects involved in this study, and they were all completely voluntary and anonymous throughout the entire study. Written informed consent to participants in this study was provided by all subjects. Written informed consent to participate in this study was provided by the participants’ legal guardian/next of kin.

## Author contributions

XL and XFW: conceptualization. XL and SW: methodology. XL, XFW, XLW, and SW: formal analysis. XL: investigation. XL and GY: data curation. XL: writing—original draft preparation. GY and XFW: writing—review and editing. XL and XLW: project administration. XFW and XLW: funding acquisition. All authors listed have made a substantial, direct, and intellectual contribution to the work, and approved it for publication.
